# Notes and News

**Published:** 1901-06-29

**Authors:** 


### Notes and News.

A new isolation hospital for Tiverton and its district has been opened recently. It has been built at a cost of £6,000. Mr. J. Siddalls was the architect.

A meeting of the Central Institute of the Tallerman Treatment was held on June 18th at the Langham Hotel, when the chairman of the occasion, Lord Llangattock, on behalf of Mr. Lewis Tallerman, handed to Commander Scott and Dr. Korttlitz, of the *Discovery*, a Tallerman apparatus for the use of the National Arctic Expedition.

A proposal is on foot to utilise some of the vacant beds at the Cardiff Infirmary for paying patients. The idea in our view is a good one, but we would suggest that the payments should be of two classes. One for those who can defray the full cost of their treatment, and the other for those who can only contribute a moderate sum towards it.

Professor Clifford Allbutt distributed the prizes to the students of the Charing Cross Hospital Medical School on Wednesday last. After the list of awards had been read out, and the successful candidates had received congratulations, Professor Allbutt delivered an address, in the course of which he much interested his hearers, who included many ladies, by various reminiscences of his early days.

The latest statistics show that there are 1,532 doctors in Denmark. The number of medical students has fluctuated according to time and circumstances. About 1840 some thirty students passed on an average their final medical examination every year. In 1846 this number, which was no doubt too large for requirements and opportunities of the period, had dwindled down to six, and for about twenty years the annual average was below twenty. After the Danish-German war there was a marked and steady increase, the figures between 1871 and 1877 ranging between forty and fifty every year. During the eighties there were, on an average, less than thirty, students every year passing their final examination, but from the year 1889 there was again a large increase, the figure that year being a record, viz., 55. Although this was considered excessive, the number went on increasing, reaching its maximum in 1898 with 81 doctors.

The Barker Anatomical Prize of the Royal College of Surgeons in Ireland is a prize of £21, offered for competition, and open to any student whose name is on the anatomical class list of any school in the United Kingdom. The preparations entered must be placed in charge of the curator before March 31st, 1902. The prize is offered for a dissection showing the distribution in the head and neck of the 7th, 8th, 9th, 10th, 11th, and 12th cranial nerves. The conditions under which the competition is to be carried out may be obtained from the curator of the Museums, Royal College of Surgeons, Dublin.

The sanitary authorities of Copenhagen have recently had printed a statement pointing out the great danger of infection from persons suffering from tuberculosis, and urging people to have rooms, wearing apparel, &c., disinfected in cases of deaths from tuberculosis, which disinfection is done free of charge. It is also strongly recommended when persons suffering from tuberculosis of the lungs leave a residence, that they should have the rooms, with their contents, disinfected before any newcomer takes possession of them. This, too, is done free of charge, when people cannot afford to do it themselves.

An open-air sanatorium for consumption will shortly be opened at Wokingham by the London Open-air Sanatorium Association. The scheme is being financed by Messrs. Weiner, Beit and Co., who will receive no interest on their money until working expenses have been paid, and then only at the rate of 2½ per cent., so that the action is practically philanthropical on the part of this great South African firm. The sanatorium is for the use of moderately well-to-do persons, who will be charged £3 3s. per week. No doubt this will be a boon to a very large class of persons, but there is a yet larger class unprovided for, and that is the class which cannot afford more than £1 1s. per annum.

The Danish sanatorium for consumptive patients, beautifully located on the borders of the Veile Fjord, was opened about a year ago. The influx of patients has been large and continuous, the number of patients averaging between 90 and 100. During the first ten months the total number of "Kurgöste" amounted to 206, of whom 8 had twice frequented the sanatorium, 117 had again left, and 89 were still there at the end of the year. The sanatorium has cost about 670,000kr.; the receipts amounted to 185,000kr.; and the expenditure to 153,000kr. The surplus, 32,000kr., was applied to various writings-off, and no dividend was paid. The sanatorium is run on business lines.

In consequence of the attention which has of late been drawn to the conditions amid which the manufacture of ice creams is often carried on, the Grimsby sanitary authority lately directed that an investigation should be made into the circumstances of the manufacture, storage, and distribution of these delicacies in that town. On the whole, except in one instance where the drain was choked, no serious sanitary defects were discovered. In several instances, however, the processes of cooling and freezing were carried on amid surroundings which were far from what they ought to be, and the advantage of having the business carried on under inspection was fully demonstrated, many reforms being at once introduced when their importance was pointed out by the inspector.

